# Targeting Anti-Inflammatory Treatment Can Ameliorate Injury-Induced Neuropathic Pain

**DOI:** 10.1371/journal.pone.0057721

**Published:** 2013-02-28

**Authors:** Katsuyuki Iwatsuki, Tetsuya Arai, Hideyuki Ota, Shuichi Kato, Tadahiro Natsume, Shigeru Kurimoto, Michiro Yamamoto, Hitoshi Hirata

**Affiliations:** Department of Hand Surgery, Nagoya University Graduate School of Medicine, Showa-ku, Nagoya, Japan; University of Medicine & Dentistry of NJ - New Jersey Medical School, United States of America

## Abstract

Tumor necrosis factor-α plays important roles in immune system development, immune response regulation, and T-cell-mediated tissue injury. The present study assessed the net value of anti-tumor necrosis factor-α treatment in terms of functional recovery and inhibition of hypersensitivity after peripheral nerve crush injury. We created a right sciatic nerve crush injury model using a Sugita aneurysm clip. Animals were separated into 3 groups: the first group received only a skin incision; the second group received nerve crush injury and intraperitoneal vehicle injection; and the third group received nerve crush injury and intraperitoneal etanercept (6 mg/kg). Etanercept treatment improved recovery of motor nerve conduction velocity, muscle weight loss, and sciatic functional index. Plantar thermal and von Frey mechanical withdrawal thresholds recovered faster in the etanercept group than in the control group. On day 7 after crush injury, the numbers of ED-1-positive cells in crushed nerves of the control and etanercept groups were increased compared to that in the sham-treated group. After 21 days, ED-1-positive cells had nearly disappeared from the etanercept group. Etanercept reduced expression of interleukin-6 and monocyte chemotactic and activating factor-1 at the crushed sciatic nerve. These findings demonstrate the utility of etanercept, in terms of both enhancing functional recovery and suppressing hypersensitivity after nerve crush. Etanercept does not impede the onset or progression of Wallerian degeneration, but optimizes the involvement of macrophages and the secretion of inflammatory mediators.

## Introduction

Tumor necrosis factor (TNF)-α plays important roles in immune system development, immune-response regulation, and T-cell-mediated tissue injury. The immune response to injury coordinates host defense and tissue repair, but also has the inherent capacity to significantly damage host tissues [1]. TNF-α antagonists such as infliximab, etanercept, and adalimumab are indicated for the treatment of refractory rheumatic diseases and inflammatory bowel diseases. In fact, use of these drugs has improved outcomes in the treatment of rheumatoid arthritis, in terms of both symptom severity scores and quality of life measures [2].

After nerve injury, TNF-α is upregulated in activated Schwann cells, macrophages, and other components of the peripheral nervous system. TNF-α has been shown to act as the initiator of Wallerian degeneration (WD) by activating resident Schwann cells and facilitating macrophage recruitment to the injury site [3].

Despite these crucial roles of TNF-α in peripheral nerve repair and regeneration, previous studies have also suggested deleterious effects of TNF-α. For instance, TNF-α released by autoreactive T cells and macrophages may induce immune-mediated demyelinating neuropathies. The proposed pathogenesis of TNF-α-associated neuropathies includes both T-cell and humoral immune attack against peripheral nerve myelin, vasculitis-induced nerve ischemia, and inhibition of signaling support for axons [4]. In fact, experimental results have demonstrated that intraneural injection of TNF-α produces predominantly axonal damage of the sciatic nerve [5]. TNF-α mediates rapid activation of injury-induced binding of nuclear factor (NF)-κB to DNA in Schwann cells, and these events are associated with inhibition of post-injury axonal sprouting [6].

Taken together, TNF-α appears to represent a two-edged sword. In fact, the merits of TNF-α-targeting treatments remain contentious. For example, Chen et al. [7] showed that TNF-α promotes functional motor recovery in crushed peripheral nerves. In contrast, many recent studies have reported that blocking TNF-α prevents nerve degeneration and promotes nerve regeneration, and motor and sensory functional recovery [8], [9]. Similarly, evidence has been accumulating indicating that inhibition of TNF-α could reduce inflammatory demyelination in various neuropathies, and TNF-α-knockout mice exhibit nerve preservation after WD induction. These reports strongly imply the central involvement of TNF-α in axonal degradation during WD [10]. In addition, several researchers have successfully demonstrated that selective inhibition of soluble TNF-α is beneficial even in the central nervous system. For example, Branbilla et al. [11] demonstrated enhanced functional recovery after experimental autoimmune encephalomyelitis, while Chio et al. [12] and Genovese et al. [13] showed neuroprotective effects after brain and spinal cord injuries.

Despite these reports of favorable effects with the use of anti-TNF-α drugs on peripheral nerve disorders, some researchers suspect that such drugs may have negative side effects for patients with peripheral neuropathies. Previous case series have suggested associations between implementation of anti-TNF-α treatment and onset or progression of peripheral nerve disorders such as Guillain-Barré syndrome, Miller Fisher syndrome, chronic inflammatory demyelinating polyneuropathy, multifocal motor neuropathy with conduction block, mononeuropathy multiplex, and axonal sensorimotor polyneuropathies [4]. Furthermore, most of these neuropathies improved over a period of months after withdrawal of the TNF-α antagonist, with or without additional immunomodulatory treatment [4], [14]. These reports sounded the alarm that anti-TNF-α therapy should be avoided in patients with pre-existing multiple sclerosis and immediately discontinued when new neurological signs and symptoms arise, pending appropriate evaluation [15].

Another issue regarding the use of TNF-α antagonists for peripheral nerve injuries or disorders is their value as a modulator of pain sensitivity. Neuropathic pain is a recognized pathological pain type where nociceptive responses persist beyond the resolution of damage to the nerve and the surrounding tissue. Satisfactory treatment of chronic pain remains elusive, and novel painkillers rarely reach the modern market [16]. Although consensus is lacking regarding the etiology of neuropathic pain, animal models of this pathology based on various nerve injury types have persistently suggested that TNF-α plays pivotal roles at both the peripheral and central levels of sensitization [17]. Likewise, Kato et al. [8] reported that anti-TNF-α therapy can attenuate neuropathic pain-related behaviors after peripheral nerve injury in a rodent model. Martini et al. [18] proposed the protection of sensory nerve fibers from macrophage attack as a challenging paradigm for the development of putative treatment approaches. In this context, knowledge of the molecular mechanisms underlying Schwann cell-macrophage interactions under pathological conditions is an important prerequisite to developing effective treatment strategies for pain problems associated with peripheral nerve disorders.

WD classically refers to the degeneration of axons distal to a lesion site. WD, the self-destructive set of cellular and molecular processes by which degenerating axons and myelin are cleared after injury, is initiated by macrophages and Schwann cells [1]. Regulating WD is one of the targets in treating central and peripheral nerve disorders and pain [19]. The molecular mechanisms implicated in axonal regeneration and pathfinding after injury are complex, and take into account cross-talk between axons and glial cells, neurotrophic factors, extracellular matrix molecules and their receptors [20]. These varied mechanisms are still not completely understood [21]. Many studies have been made with the help of Wallerian degeneration slow (Wld^S^) mice [22], [23], [24], [25], [26]. The phenotype in this model is attributed to overexpression of a chimeric protein, Wld^S^, which contains the full-length nicotinamide adenine dinucleotide synthetic enzyme, nicotinamide mononucleotide adenylyl-transferase-1 (Nmnat-1) [27], [28], [29]. Nmnat1 has been reported to protect the axon through NAD-dependent deacetylase sirtuin 1 or local nicotinamide adenine dinucleotide synthesis in neuritis, but Nmnat1 is considerably weaker than Wld^S^ [30], [31], [32]. In this mouse mutant, axon stumps distal to the lesion site survive ten times longer than axons in wild-type animals [33], [34]. This protein is being studied as a treatment target for central and peripheral nerve disorders, such as trauma [35], Parkinson’s disease [36], and Charcot-Marie-Tooth disease [37].

Molecular inflammatory mediators such as interleukin (IL)-1, IL-6, IL-10, TNF-α and NF-κB, the complement system and arachidonic acid metabolites have been shown to modulate these processes. In addition, Schwann cells of the WD following axonal injury can produce TNF-α and IL-1 [38]. NF-κB activation is important for TNF-α secretion from Schwann cells and may play a key role in triggering positive-feedback loops for IL-6 expression [39]. Neurons can synthesize and express molecular inflammatory mediators, and these cytokines may participate in neuronal communication [40], [41], [42].

The release and activity of TNF-α and other potentially damaging cytokines are controlled at multiple levels to prevent unrestrained collateral tissue damage that can disable, or even kill, the host [43]. As systemic reactions, humoral mechanisms that restrain or inhibit these damaging responses include glucocorticoid hormones, soluble cytokine receptors, IL-10, transforming growth factor-β and other anti-inflammatory cytokines. Activation of cholinergic receptors is also known to regulate immune system activity [38], [44].

Direct modulation of voltage-gated sodium channel (Nav) expression by IL-1 and IL-6 is produced by macrophages via activation of purinergic receptor P2X, ligand-gated ion channel (P2RX) 7, and direct activation through a signaling pathway that involves TNF-α acting on TNF receptors. Cytokines produced by inflammatory and glial cells change neuronal excitability and this link contributes directly to the development of intractable pain.

The purpose of the present study was to assess the net value of the anti-TNF-α treatment in terms of functional recovery and inhibition of hypersensitivity after peripheral nerve crush injury using a rat model. In addition, we attempted to elucidate the molecular mechanisms involved, with special emphasis on the effects of TNF-α on macrophage behavior and IL-6 production during WD and axonal regeneration at the injured nerve.

## Materials and Methods

### Rat Model of Sciatic Nerve Injury

All experimental protocols and animal maintenance procedures were approved by the Animal Ethics Research Committee of Nagoya University (Permit number: 24007), and were in accordance with the Animal Protection and Management Law of Japan (No. 105) and the Ethical Issues of the International Association for the Study of Pain [45].

A total of 138 sciatic nerves of Lewis rats (body weight, approximately 250 g) were used. Animals were anesthetized with an intraperitoneal injection of 5% pentobarbital. Animals were then separated into 3 groups. The first group received only a skin incision (sham group, n = 46). The second group received a nerve crush injury and an intraperitoneal injection of vehicle (solvent saline) (control group, n = 46). The third group received a nerve crush injury and injection of etanercept (Takeda, Osaka, Japan) (etanercept group, n = 46). In both the control and etanercept groups, the right sciatic nerves were dissected from the surrounding tissues and nerve crush injuries [46] were inflicted using a Sugita aneurysm clip (Mizuho Ikakogyo, Tokyo, Japan). The clip was applied for 5 min with approximately 1.5 N of holding force. In the etanercept group, etanercept was administered once intraperitoneally at 6 mg/kg on the day of operation [9].

### Electrophysiological Evaluation

According to previous studies, sciatic nerve crush using a Sugita clip for 5 min inevitably leads to complete WD in rats, with spontaneous recovery within 6 weeks. We therefore conducted electrophysiological assessments on days 7, 21 and 35 after crush injury to measure motor nerve conduction velocities (MCVs) of the tibial nerve in the control, etanercept, and sham groups.

Compound muscle action potentials (CMAPs) of the tibialis anterior muscle were measured at room temperature (24°C). Two stainless steel monopolar recording electrodes (H537A; Nihon Kohden, Tokyo, Japan) were placed on the center of the belly of the tibialis anterior muscle after exposing the muscle. The sciatic nerve was exposed and a bipolar stimulating electrode (UM2-5050; Nihon Kohden) was placed around the nerve at the level of the sciatic notch. Electrical pulses (supramaximal; duration, 100 ms; frequency, 1 Hz; square wave) were applied with an isolator (SS-201J; Nihon Kohden) connected to an electronic stimulator. CMAPs were recorded to estimate electrophysiological function and evaluate MCV [47], [48].

### Wet Muscle Weight Measurement

After euthanasia, tibialis anterior muscle was dissected free from the origin and insertion, and immediately weighed while still wet (n = 5 per group). Tibialis anterior muscle weight was then calculated as a percentage of total body weight [48].

### Behavioral Tests

Sensitivity to non-noxious mechanical stimuli was measured using the von Frey test. The rats stood on an elevated platform made of a wide-gauge wire mesh. The von Frey hair was inserted from below, up through the holes in the mesh, and poked through the undersurface of a hind paw. At threshold, the animal quickly responded by flicking its paw away from the hair.

Thermal hyperalgesia was assessed by measuring hind paw withdrawal latency in response to radiant heat using a plantar test apparatus (Ugo Basile, Comerio, Italy). Each rat was placed into a compartment enclosure on a glass surface. A mobile heat source was then positioned under the plantar surface of the hind paw and activated with a light beam [49], [50]. Results were calculated as a ratio compared to values in the sham group.

### Sciatic Nerve Function

Sciatic functional index (SFI) [51], an index of the functional condition of rat sciatic nerve function based on measurements made from walking tracks, was measured using a CatWalk XT 8.0 automated quantitative gait analysis system (Noldus, Wageningen, the Netherlands) [52], [53]. To obtain an overall index of the degree of normal function, the 4 variables measured on each side were entered into the following formula [51]:
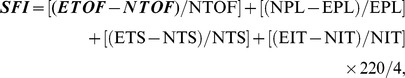
where N is normal, E is experimental, TOF is the distance to the opposite foot, PL is print length, TS is total toe spreading, and IT is the distance between intermediary toes.

### Real-time Polymerase Chain Reaction

Total RNA was isolated from crushed sciatic nerves (n = 5 per group) and L5 dorsal root ganglia (DRGs) after crush injury using an RNeasy Lipid Tissue Mini Kit (Qiagen, Valencia, CA), and complementary DNA was made using High-Capacity RNA to cDNA Master Mix (Applied Biosystems, Foster City, CA). Specific primers for TNF-α, IL-6, IL-1β, monocyte chemoattractant protein (MCP)-1, P2RX7 and voltage-gated sodium channels from Nav1.3, 1.8, 1.9 and TaqMan probes (Applied Biosystems) were used. Expression levels of TNF-α, IL-6, IL-1β, MCP-1 and P2RX7 were investigated at the crushed nerves and expression levels of sodium channels were investigated at the DRG.

Samples were subjected to 40 cycles of amplification at 95°C for 15 s and 60°C for 1 min, after holding at 50°C for 2 min and at 95°C for 10 min, and mRNA expression differences among the 3 groups were determined by calculating Δ*C*_T_s (*C*_T_ for each target minus *C*_T_ for β-actin for each sample).

### Enzyme-linked Immunosorbent Assay for TNF-α and IL-6

A rat TNF-α immunoassay system (RayBio, Norcross, GA) and an IL-6 immunoassay system (Abnova, Taipei, Taiwan) were used. Crushed sciatic nerves (n = 5 per group) at 2, 7, 21, and 35 days after crush injury were homogenized in Tris-buffered saline containing 1% Nonidet P-40, 10% glycerol, and protease inhibitors. Samples were diluted to the same protein concentration. Results were calculated as a ratio compared to values in the sham group.

### Immunohistochemistry

Rats were anesthetized as described above and perfused transcardially with fresh 4% paraformaldehyde. Crushed sciatic nerves were removed and postfixed in 4% paraformaldehyde overnight. Tissues were embedded in paraffin, then 10-µm sections were deparaffinized with xylene and rehydrated in graded ethanol. For antigen retrieval, all sections were incubated with protease for 60 min and blocked with 10% goat serum in 0.01-M phosphate-buffered saline with 0.3% Triton X-100 for 2 h at room temperature. Sections were incubated with mouse anti-rat ED-1 (1∶100; Abnoba, Taipei, Taiwan) overnight at 4°C, and then goat anti-mouse Alexa 488 fluorescent antibody (1∶1,000 A11029; Life Technologies, Gaithersburg, MD) for 1 h.

### Statistical Analysis

Statistical analyses were performed using two-way repeated-measures analysis of variance to consider the kinetics-based analyses of the control and etanercept groups. Differences between the three groups were calculated using one-way analysis of variance and the Tukey test for post hoc comparisons when significance was determined by analysis of variance. Values of *p*<0.05 were considered statistically significant.

## Results

### Electrophysiological Evaluation after Nerve Crush Injury

**Figure 1:**
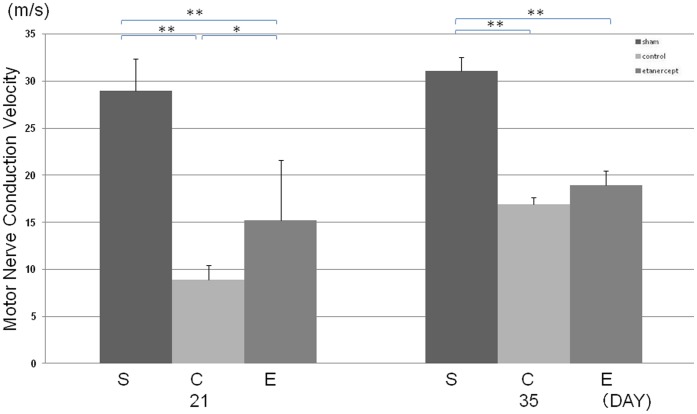
Electrophysiological evaluation after nerve crush injury. Motor nerve conduction velocity of the tibialis anterior muscle was significantly longer in the control group than in the etanercept and sham groups at 21 days post-crush injury (**p<0.01; *p<0.05).

On day 7, MCVs were not evoked for either the control group or the etanercept group. The results of electrophysiological assessments on days 21 and 35 after crush injury are shown in Figure 1. On day 21, MCVs were significantly slower for the control and etanercept groups than for the sham group, and MCV for the control group was significantly slower than that of the etanercept group. On day 35, MCV of the control group also recovered and significant differences were no longer evident between control and etanercept groups.

### Effect of Etanercept on Wet Muscle Weight Measurement after Nerve Crush Injury

**Figure 2:**
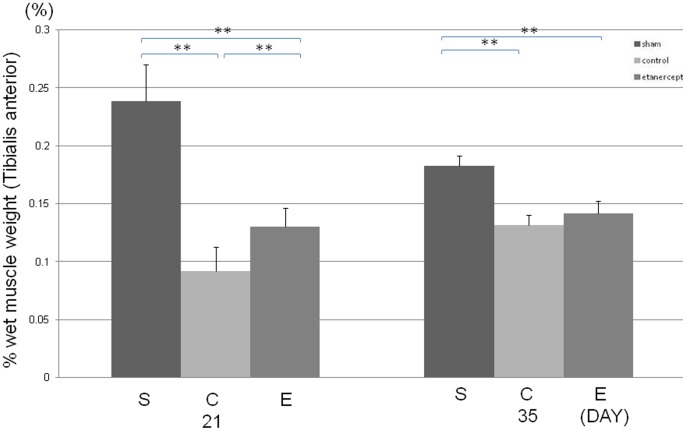
Effect of etanercept on wet muscle weight measurement. Control group showed a significantly lower percentage of wet muscle weight than the etanercept group on day 21 (**p<0.01).

The etanercept and control groups exhibited significantly lower percentage wet muscle weights than the sham group on days 21 and 35 (Fig. 2). Furthermore, the control group exhibited a significantly lower percentage wet muscle weight than the etanercept group on day 21. However, on day 35, no significant difference was observed between the etanercept and control groups.

### Effects of Etanercept on SFI after Nerve Crush Injury

**Figure 3:**
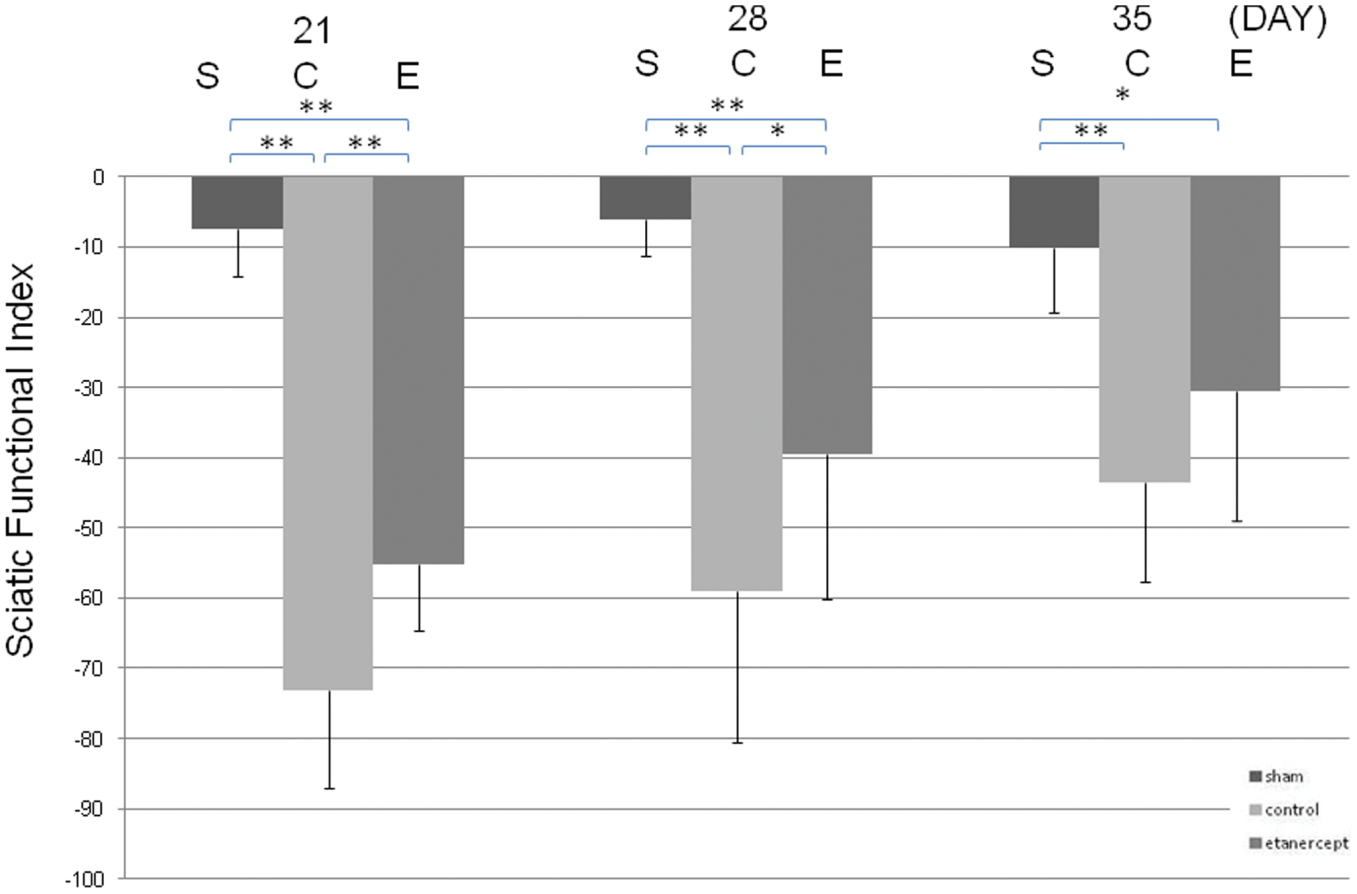
Effects of etanercept on sciatic functional index. Variations in sciatic functional index between groups after nerve crush injury. Sciatic functional index differed significantly between the 3 groups (**p<0.01; *p<0.05).

Although paresis of the right hind paw was observed in the control and the etanercept groups, SFI differed significantly between these 2 groups. Etanercept treatment significantly ameliorated sciatic nerve function on days 21 and 28 after crush injury (Fig. 3).

### Effects of Etanercept on Behavioral Tests after Nerve Crush Injury

**Figure 4:**
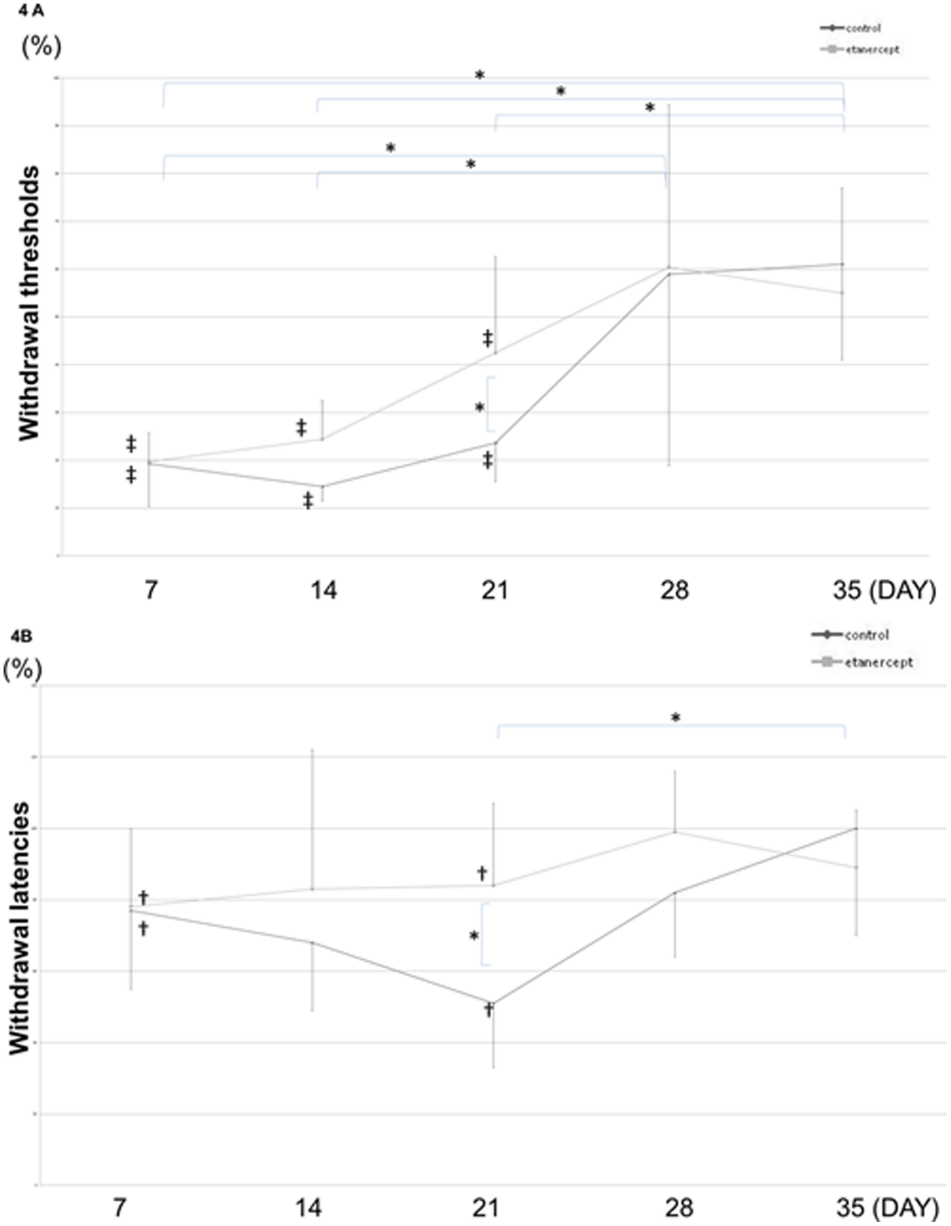
Effects of etanercept on behavioral tests. **A)** von Frey test **B)** Plantar test The von Frey mechanical withdrawal threshold and plantar thermal withdrawal threshold after crush operation. Control and etanercept groups exhibited mechanical hyperalgesia and thermal hyperalgesia on day 21, with effects attenuated by etanercept therapy (**p<0.01; *p<0.05; ‡*p*<0.01; †*p*<0.05, compared to sham group).

Mechanical and plantar thermal withdrawal thresholds were measured after nerve crush injury. The von Frey test clearly demonstrated mechanical hyperalgesia in both the control and etanercept groups, but mechanical hyperalgesia resolved faster in the etanercept group than in the control group (Fig. 4A). The plantar test likewise showed thermal hyperalgesia in both control and etanercept groups. Again, thermal hyperalgesia was attenuated by etanercept therapy at 21 days after crush injury, but persisted in the control group (Fig. 4B).

### Effects of Etanercept on mRNA Expression after Nerve Crush Injury

**Figure 5:**
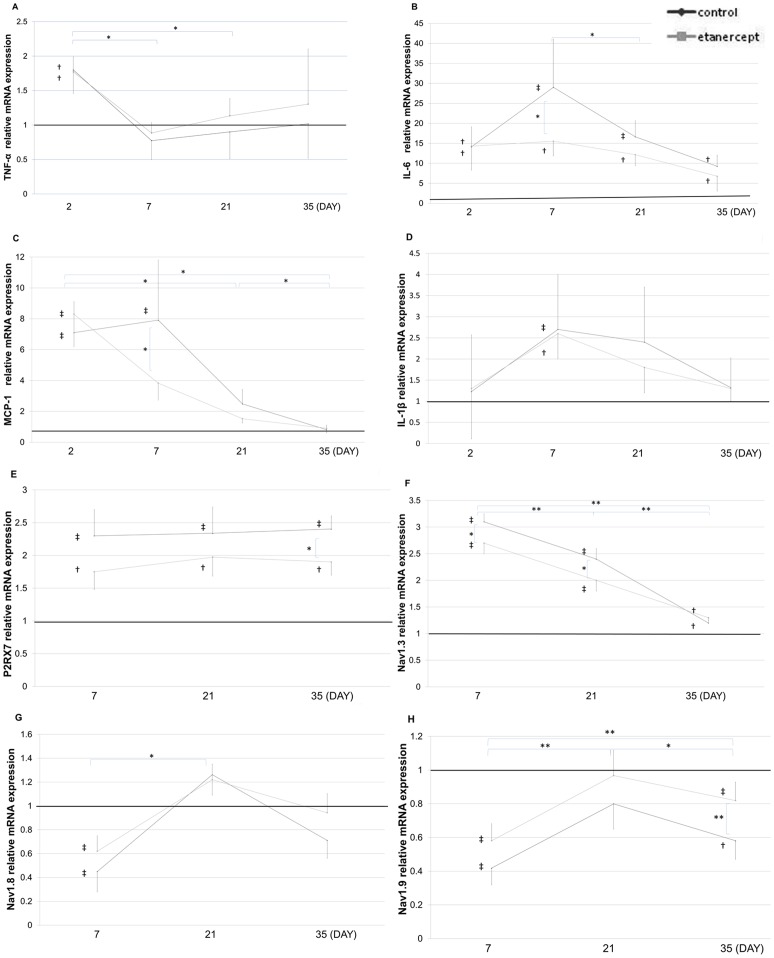
Effect of etanercept on mRNA expressions. The baseline was drawn at a value of 1 (average for the sham group) (**p<0.01; *p<0.05; ‡*p*<0.01; †*p*<0.05, compared to sham group). **A)** Expressions of TNF-α mRNA at the crushed nerve. **B)** Expression of IL-6 mRNA at the crushed nerve. **C)** Expression of MCP-1 mRNA at the crushed nerve. **D)** Expression of IL-1 mRNA at the crushed nerve. **E)** Expression of P2RX7 mRNA at the crushed nerve. **F)** Expression of Nav1.3 mRNA at the L5 DRG. **G)** Expression of Nav1.8 mRNA at the L5 DRG. **H)** Expression of Nav1.9 mRNA at the L5 DRG.

The mRNA levels of TNF-α and IL-6 after crush injury were determined by real-time polymerase chain reaction. Expression of TNF-α mRNA in the control and etanercept groups was increased on day 2, but the difference between the 2 groups was not significant, and expression returned to baseline levels by day 7 (Fig. 5A). Expressions of IL-6 mRNA were significantly higher in the control and etanercept groups and remained elevated over 35 days, but etanercept treatment reduced expression of IL-6 mRNA compared to the control group on day 7 (Fig. 5B). Expression of MCP-1 mRNA was significantly higher in the control and etanercept groups on day 2, but etanercept treatment reduced expression of MCP-1 mRNA on days 7 (Fig. 5C). Expression of IL-1β mRNA was upregulated on day 7 after nerve crush, but no significant differences between the control and etanercept groups were identified (Fig. 5D). Expression of P2RX7 mRNA was upregulated after crush injury, and etanercept reduced the P2RX7 expression (Fig. 5E). Crush injury appeared to change the types of sodium channel expressed at the DRG, and etanercept reduced these changes (Fig. 5F–H).

### Effects of Etanercept on TNF-α and IL-6 Expression after Nerve Crush Injury

**Figure 6:**
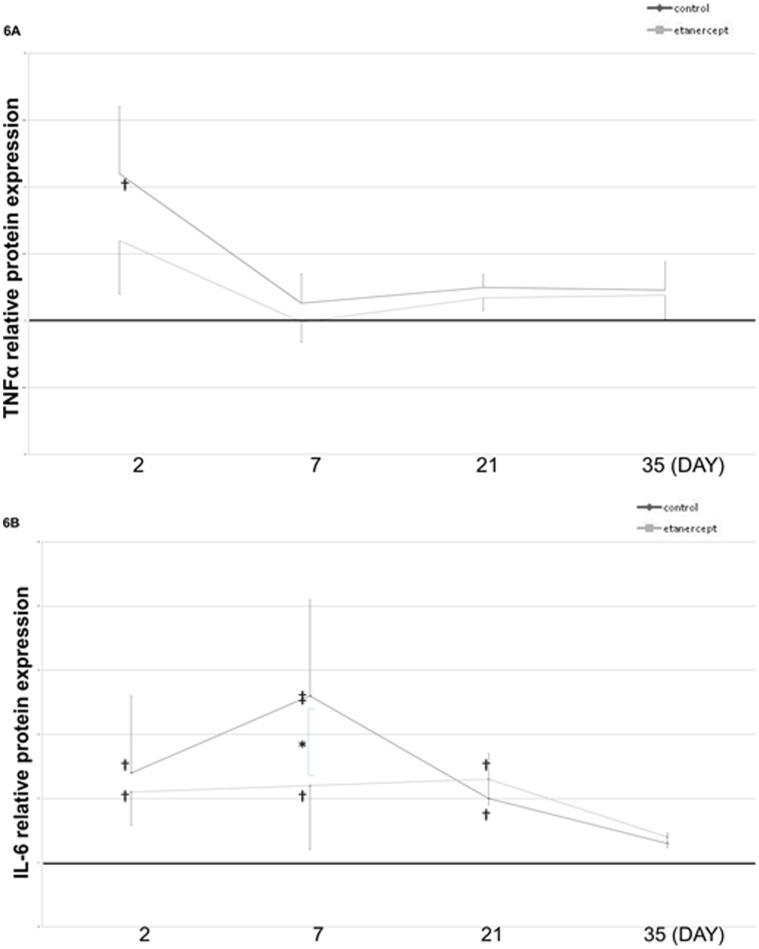
Effects of etanercept on TNF-α and IL-6 expression. Protein levels of TNF-α (**A**) and IL-6 (**B**) were determined in homogenized sciatic nerves. TNF-α levels were significantly increased 2 days after crush injury, but IL-6 upregulation lasted more than 21 days. Etanercept inhibited upregulation of IL-6 levels (**p<0.01; *p<0.05; ‡*p*<0.01; †*p*<0.05, compared to the sham group).

Protein levels were determined in homogenized sciatic nerves by enzyme-linked immunosorbent assay after crush injury. In nerve crush injury groups, TNF-α levels were significantly increased on day 2, but no significant differences were evident between the control and etanercept groups, and both groups returned to baseline levels by day 7. After 7 days, significant differences were apparent between the three groups. However, IL-6 upregulation lasted more than 21 days (Fig. 6A) and returned to baseline by day 35. Etanercept inhibited upregulation of IL-6 on day 7 after crush injury (Fig. 6B).

### Immunostaining for Macrophage Marker ED-1 after Nerve Crush Injury

**Figure 7:**
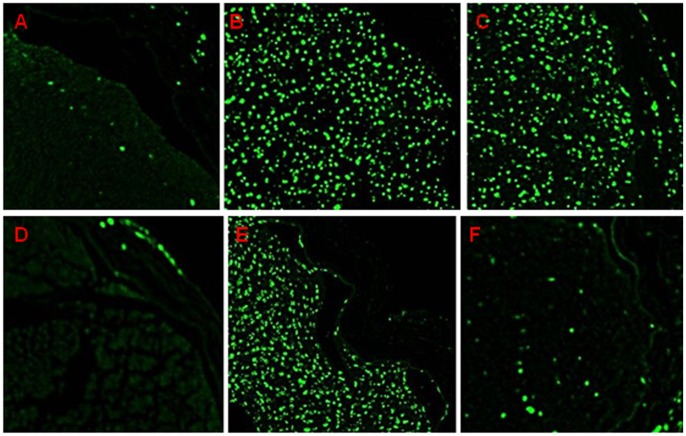
Immunohistochemical staining for macrophage marker ED-1 in crushed sciatic nerve sections. **A)** Sham-treated 7 days after surgery; **B)** control 7 days after surgery; **C)** etanercept-treated 7 days after surgery; **D)** sham-treated 21 days after surgery; **E)** control 21 days after surgery; and **F)** etanercept-treated 21 days after surgery. By 21 days, ED-1-positive cells had nearly disappeared from the etanercept group.

On day 7 after crush injury, ED-1-positive cells were observed in both control and etanercept groups. This means that macrophages were recruited from the periphery and induced WD by carrying out the removal of myelin debris while Schwann cell proliferation was concomitantly enhanced. After 21 days, ED-1-positive cells had almost disappeared in the etanercept group, whereas the control group still showed abundant ED-1-positive cells within nerve fascicles (Fig. 7).

## Discussion

After nerve injury, TNF-α is upregulated in activated cells of the central and peripheral nervous systems. These activated cells are considered to be glia (including Schwann cells), mast cells, endothelial cells, perineurial cells, and both resident and hematogenous macrophages [19].

TNF-α has been shown to act as the initiator of WD by activating resident Schwann cells and facilitating macrophage recruitment to the injury site [3]. TNF-α promotes functional motor recovery in crushed peripheral nerves [7]. Even though TNF-α is needed in peripheral nerve repair and regeneration, anti-TNF-α therapy is also being studied for application in nerve disorders [12]. Anti-TNF-α therapy prevents nerve degeneration and promotes nerve regeneration, and motor and sensory functional recovery [8], [9]. These seemingly contradictory reports suggest the complexity and significance of TNF-α involvement in axonal degradation during WD.

Etanercept is a bioengineered dimeric fusion protein consisting of a soluble human 75-kDa TNF-α receptor and the Fc protein of immunoglobulin G1. This protein shows a greatly extended half-life in the bloodstream compared to naturally occurring soluble TNF receptors [54], and can neutralize both human and rat TNF-α [6], [8], [12]. As mentioned above, TNF-α inhibition can be a two-edged sword. Even pharmaceutical companies distributing anti-TNF-α drugs are proving hesitant to expand the indications to cover neurological problems, instead recommending careful use of these drugs, particularly for patients with demyelinating conditions. The present study therefore aimed to evaluate the net effect of etanercept on axonal regeneration after nerve crush injury.

Scholz and Woolfe [55] recently pointed out many features of neuroimmune disorders in neuropathic pain and suggested the possibility that blockade of reciprocal signaling pathways between neuronal and non-neuronal cells offers new opportunities for more successful pain management. According to Scholz and Woolfe, two different pathways are involved in the sensitization process of nociceptors: direct modulation of Nav expression by IL-1 and IL-6 produced by macrophages via activation of homodimeric P2RX7; and direct activation through a signaling pathway that involves TNF-α acting on TNF receptor 1 [55], [56], [57]. Isoforms of P2RX2, PR2X3, P2RX4, and P2RX7 are considered to participate in pain perception [58]. These receptor subtypes thus represent promising targets for pain management.

Nociceptive activity is further modulated by activation of the dimeric purinergic receptor comprising P2RX2 and P2RX3, which is mediated in part by voltage-gated sodium channels. P2RX7 dysfunction results in substantially blunted hypersensitivity to mechanical and thermal stimuli in models of neuropathic pain, presumably because of reduced release of inflammatory cytokines from macrophages and microglia [59]. Crush injury changes the expression levels of cytokines, P2RX, and sodium channels, and after nerve injury the changes of these expressions are similar to those in neuroimmune disorders. The present study aimed to investigate expression changes of these chemical mediators and macrophage activity after anti-TNF-α treatment.

According to George, Sawada, and Kato [9], [60], [61], who separately studied spatiotemporal expression patterns of TNF-α during WD, TNF-α expression is enhanced promptly after nerve injury in the distal stump, peaking between day 1 and day 3 post-injury, and returning to baseline levels as early as day 7. This temporal expression pattern was reconfirmed in the present study, implying that TNF-α plays roles during the early phase of WD. According to previous studies, TNF-α plays multiple roles in triggering WD. Liefner et al. [62] demonstrated that TNF-α can induce macrophage recruitment from the periphery even without myelin damage or phagocytosis. TNF-α is linked to the development of abnormal blood-nerve barrier permeability [63], monocyte recruitment or residential macrophage activation, and coordination of these inflammatory events with demyelination. Infiltrating macrophages also contribute to the removal of myelin debris, and enhance concomitant Schwann cell proliferation, probably by releasing soluble mitogenic factors [64]. Taking these factors into consideration, we decided to use bolus intraperitoneal injection at the time of nerve crush.

The present study quantified TNF-α expression level in the nerve segment distal to the crush site. As demonstrated above, no significant differences in TNF-α expression were seen at the RNA and protein levels, while significant suppression of IL-6 expression was observed at both RNA and protein levels. This indicates that etanercept did not affect TNF-α production, but instead inhibited its biological function. In fact, immunohistochemical staining for the macrophage marker ED-1 clearly demonstrated that macrophage infiltration inside nerve fascicles took place on day 7 not only in the control group, but also in the etanercept group. This indicates that a single bolus intraperitoneal injection of etanercept does not blockade the onset and progression of WD. However, a marked difference in macrophage distribution between groups was seen on day 21. According to Sommer et al. [65], macrophages are involved in generation of neuropathic pain and the establishment of hyperalgesia following chronic constrictive nerve injury in which Wallerian-like degeneration and macrophage activation take place. Localized WD associated with macrophage influx and proinflammatory cytokine production thus appears to be a critical factor in the development of hyperalgesia in animal models of neuropathic pain. In fact, Sommer and Schafers [66] subsequently emphasized the possibility that macrophage invasion and TNF-α production influence the development of thermal hyperalgesia and that regenerative activity is linked to mechanical allodynia in peripheral mononeuropathy.

TNF-α lies upstream of matrix metalloproteinase-9 in the pathway of macrophage recruitment to injured peripheral nerves [67], [68], and stimulates myelin degradation in the distal nerve fragment [69]. In addition, TNF-α also induces other chemotactic factors, such as MCP-1 [70]. MCP-1 plays essential roles in the recruitment of monocytes into lesions of spinal cord contusion and neuroinflammation [71], [72], [73]. In the present study, etanercept reduced MCP-1 expression on days 7 and 21, supporting the results of macrophage recruitment by immunostaining. Endogenous MCP-1, which displays chemotactic activity for monocytes, also plays an important role in the full expression of neuropathic pain. Treatment with anti-MCP-1 neutralizing antibody effectively attenuates neuropathic pain [74], [75] following chronic constriction injury of the sciatic nerve [76] and L5 spinal nerve ligation [77].

We consider this a significant observation from a therapeutic perspective. This is because axonal regeneration never takes place without removal of myelin debris (which can strongly repel growing axons) or without formation of a so-called Schwann cell column within the basal lamina, and infiltrating macrophages are responsible for the whole process, as discussed above. Any drug that can completely suppress macrophage infiltration should thus be detrimental to nerve regeneration. We also believe that the prolonged presence of activated macrophages inside the nerve fascicle would also be detrimental to the growth and maturation of regenerating axons, as both electrophysiological and walking track analyses clearly demonstrated significantly quicker functional recovery in the etanercept group.

Macrophage recruitment is an important component of nerve growth factor (NGF) synthesis and of sensory neuron maintenance and axonal regrowth [78]. However, over the past decade, considerable evidence has accumulated indicating that NGF is also a strong mediator of peripheral pain, particularly in chronic pain status [79].

Sensitization to endogenous TNF-α may be essential for the development and maintenance of neuropathic pain [80]. The present study assessed the effects of etanercept on pain status. As demonstrated clearly with both von Frey and plantar thermal tests, both mechanical and thermal hypersensitivity occurred regardless of treatment on Day 7, when macrophage infiltration takes place. However, hypersensitivity improved more rapidly in the etanercept group than in the control group, consistent with the earlier disappearance of infiltrating macrophages.

Etanercept also reduced expression levels of P2RX7. P2RX7 may be an appealing target for pharmacological intervention [56], [81], since this protein may represent a critical communication link between the nervous and immune systems. P2RX7 was originally described in cells of hematopoietic origin, including macrophages, microglia, and certain lymphocytes [82]. P2RX7-knockout animals thus show reductions in IL-1, IL-6, and MCP-1 [59].

Although some reports have examined IL-6 expression after nerve injury [83], [84], none have reported serial changes in IL-6 expression in crushed nerves over 5 weeks, and none have reported the role of IL-6 in WD. The present study demonstrated prolonged up-regulation of IL-6 expression even at 35 days after crush injury, but significantly reduced by etanercept. IL-6 belongs to a group of cytokines that control inflammatory responses, in part by regulating the synthesis and release of additional cytokines. IL-6 is a pleiotropic cytokine with robust pro-inflammatory activity. Increased IL-6 expression has been demonstrated during peripheral nerve injury-induced WD following hypoglossal nerve axotomy [83]. In addition, IL-6 was found to be secreted by macrophages and denervated Schwann cells, and is released into the distal nerve stump after nerve injury [85]. IL-6 administration is able to stimulate neurotrophin-dependent neurite outgrowth in cultured dorsal root ganglions [86]. These findings seem to identify IL-6 as an important factor in the onset and progression of axonal regeneration. In addition, IL-6 was recently reported to be involved in neuropathic pain [87]. According to Ohtori et al. [88], epidural administration of an anti-IL-6 receptor monoclonal antibody, tocilizumab, onto the spinal nerve reduced radicular leg pain, numbness, and low back pain without causing any appreciable adverse events. The precise function of IL-6 in WD and axonal regeneration remains to be elucidated [10], but considering the fact that the etanercept group in which IL-6 expression was downregulated showed swifter functional recovery and normalization of pain sensitivity, regulation of IL-6 expression during nerve regeneration may hold promise as a therapeutic strategy [40].

The possibility remains that the whole reaction was affected by more global pain modulating mechanisms [40]. The nervous system can synthesize and express TNF-α, and these cytokines may participate in neuronal communication [41], [42]. This communication is bi-directional, because cytokines can activate hypothalamic-pituitary release of glucocorticoids and, in turn, glucocorticoids suppress further cytokine synthesis [89]. In addition, cells of the immune system can produce neuropeptides, acetylcholine and other neurotransmitters. Acetylcholine interacts with cytokine-producing macrophages in the red pulp and marginal zone to suppress TNF-α release [90]. A prototypical anti-inflammatory neural mechanism is known as the inflammatory reflex [40], [91], [92]. Action potentials arising in the brainstem are transmitted in the cholinergic vagus nerve to terminate in the celiac ganglion, the site of origin of the adrenergic splenic nerve. Electrical stimulation of either the vagus nerve above the celiac ganglion or the splenic nerve itself significantly inhibits production and release of TNF-α into the circulation by macrophages in the red pulp and marginal zone of the spleen [93]. The spleen is a major organ for the anti-inflammatory effects of efferent vagus nerve signals [1], [90].

The mechanism of the inflammatory reflex requires the α7 nicotinic acetylcholine receptor (α7nAChR), a ligand-gated ion channel expressed on macrophages, neurons and other cells [94], [95]. Deleting α7nAChR from isolated macrophages impairs the ability of acetylcholine to suppress TNF-α and other cytokines [1]. The importance of the interaction between the nervous system and immune system signaling has been demonstrated in the development of pain. Cytokines produced by inflammatory and glial cells change neuronal excitability and this contributes directly to the development of intractable pain [96]. The inhibition of TNF-α may relate to the inflammatory reflex at the global reflex and regulation of cytokine networks.

While this study specifically focused on local cytokine networks, and the regulation of macrophage activities at the injury site, we are going to look at interactions between the nervous and inflammatory systems in the future.

### Conclusion

This study clearly demonstrated that bolus intraperitoneal injection of etanercept, an anti-TNF-α antibody, is useful in terms of both enhancing functional recovery and suppressing hypersensitivity after nerve crush. It should be emphasized that etanercept does not impede the onset or progression of WD, but instead optimizes the involvement of macrophages and the secretion of inflammatory mediators. Treatment protocols in terms of dosage and frequency appear to be important in preventing possible side-effects from jeopardizing the normal process of WD and axonal degeneration.
